# Atypical Balance between Occipital and Fronto-Parietal Activation for Visual Shape Extraction in Dyslexia

**DOI:** 10.1371/journal.pone.0067331

**Published:** 2013-06-25

**Authors:** Ying Zhang, Susan Whitfield-Gabrieli, Joanna A. Christodoulou, John D. E. Gabrieli

**Affiliations:** 1 Department of Brain and Cognitive Sciences, McGovern Institute for Brain Research, Massachusetts Institute of Technology, Cambridge, Massachusetts, United States of America; 2 State Key Laboratory of Brain and Cognitive Science, Institute of Biophysics, Chinese Academy of Science, Beijing, China; University of Muenster, Germany

## Abstract

Reading requires the extraction of letter shapes from a complex background of text, and an impairment in visual shape extraction would cause difficulty in reading. To investigate the neural mechanisms of visual shape extraction in dyslexia, we used functional magnetic resonance imaging (fMRI) to examine brain activation while adults with or without dyslexia responded to the change of an arrow’s direction in a complex, relative to a simple, visual background. In comparison to adults with typical reading ability, adults with dyslexia exhibited opposite patterns of atypical activation: decreased activation in occipital visual areas associated with visual perception, and increased activation in frontal and parietal regions associated with visual attention. These findings indicate that dyslexia involves atypical brain organization for fundamental processes of visual shape extraction even when reading is not involved. Overengagement in higher-order association cortices, required to compensate for underengagment in lower-order visual cortices, may result in competition for top-down attentional resources helpful for fluent reading.

## Introduction

Developmental dyslexia is a significant impairment in reading, regardless of age, IQ, or educational opportunity [1], [2], and it is associated with atypical brain function for reading [3], [4]. The best understood cause for dyslexia is a weakness in phonological awareness [5], [6]. Phonological training, however, is more effective in improving reading accuracy than reading speed [1], [7], which suggests that other factors, such as visual deficits, may also play a causal role in preventing readers with dyslexia from efficiently extracting information from written language.

Visual deficits are frequently reported in dyslexia, although the nature and role of these deficits are less agreed upon. Using non-verbal visual stimuli such as gratings or simple geometric shapes, separate lines of evidence suggest dyslexic deficits in either sensory or attentional processes that are independent from language [8], [9]. Visual sensory deficits associated with magnocellular functions have been suggested by anatomical, physiological, and behavioral studies [9], [10], [11], but the specificity of these deficits have been challenged [12], [13], [14], [15], [16], [17]. The sensory deficit is further supported, however, by neuroimaging findings of reduced activation in individuals with dyslexia for visual motion in early visual areas [18], [19]. Aside from sensory problems, visual attentional deficits in dyslexia are suggested by impaired performance on non-verbal covert attention or visual search tasks [20], [21], [22], [23], [24]. Indeed, children with dyslexia who also have reduced visual attention spans show reduced activation in parietal areas associated with visual attention [25].

Neuroimaging studies of basic visual processing in dyslexia have focused on motion or attention, but it is unknown as to whether individuals with dyslexia extract visual shapes differently in their brains than do typical readers. Fluent and skilled reading begins with shape extraction of letters from complex text and visual environments, and an altered brain basis of visual shape extraction could compromise reading. Here, we compared brain functions between adults with and without dyslexia for basic visual extraction of a non-verbal shape from a complex background. We used functional magnetic resonance imaging (fMRI) to investigate brain activations for a simple task in which a shape (an arrow) had to be extracted from a simple or a complex visual background. Behavioral studies have reported that individuals with dyslexia are impaired at extracting shape information from complex white noise backgrounds [16], [26], [27], [28], [29]. A fundamental question is whether visual shape extraction in dyslexia is associated with altered brain functions, and if so whether such alterations occur in occipital areas associated with sensory processes, in fronto-parietal areas associated with attentional processes, or in both sensory and attentional areas.

## Materials and Methods

All participants were recruited from the Boston area and were provided written informed consent as approved by the Massachusetts Institute of Technology Institutional Review Board (IRB).

### Participants and Testing Battery

Twenty-four healthy participants, between the ages of 18–37 years and having normal or corrected-to-normal vision were included. All participants were native English speakers, had no neurological or psychiatric disorders, were not on any medication, and had no contraindications for MRI. All participants were paid $20/hour for their participation.

For all participants, cognitive abilities, reading and related skills were assessed using a battery of standardized behavioral measures. The testing battery included indices of non-verbal cognitive ability (*Kaufman Brief Intelligence Test* (KBIT) [30], or *Wechsler Abbreviated Scale of Intelligence* (WASI) [31]. Untimed reading ability was measured by the total numbers of real words and pseudowords that were read correctly (“Word Identification” and “Word Attack” from the *Woodcock Reading Mastery Test-Revised, NU* (WRMT) [32]). Timed reading abilities were measured by the total numbers of words and pseudowords that were read correctly within a time limit (“Sight Word Efficiency” and “Phonemic Decoding Efficiency” from the *Test of Word Reading Efficiency* (TOWRE) [33]). Rapid naming scores were the time in seconds needed to name a series of letters as quickly and accurately as possible (“Rapid Letter Naming,” *Comprehensive Test of Phonological Processing)* [34].

### Participant Groups and Inclusion Criteria

Eleven participants with dyslexia and thirteen people with typical reading ability (i.e., the control group) were matched based on their non-verbal cognitive abilities (Performance IQ, Table 1). On measures of cognitive ability, all participants scored within 15 points or higher of the expected mean of 100. Criteria for dyslexia were met if participants reported a previous history of reading difficulty or a related diagnosis (e.g., reading disability, dyslexia), and also earned a current standard score below the 25^th^ percentile rank based on norms from standardized tests, on at least two measures of single word or pseudoword timed or untimed reading.

**Table 1:** Behavioral Profiles for Participants with and without Dyslexia.

	Control Readers	Dyslexic Readers	T-values: Control vs. Dyslexic Readers
	Mean±SD	Mean±SD	T p
*N*	13(8 female)	11 (7 female)	
Age	22.9±3.7	24.3±5.4	0.75.46
**Behavioral Measure**			
Performance IQ	108.9±8.4	107.2±13.9	0.38.71
Rapid Letter Naming	0.61±.50	−0.29±.86	3.19.004***
WRMT – Word Identification	107.1±7.2	92.9±8.9	4.32.000***
WRMT – Word Attack	107.9±8.8	92.0±12.6	3.63.001**
TOWRE – Sight Word Efficiency	109.1±6.9	91.3±13.5	4.16.000***
TOWRE – Phonemic Decoding Efficiency	106.2±7.3	82.8±5.0	9.02.000***

* *p*<0.05, two-tailed t-test;

** *p*<0.01, two-tailed t-test;

*** *p*<0.001, two-tailed t-test.

Note: Standard scores are based on a mean of 100 and a standard deviation (SD) of 15. Note: Acronyms are as follows: WRMT for *Woodcock Reading Mastery Test – Revised NU;* TOWRE for *Test of Word Reading Efficiency. Note: Participants completed either the Kaufman Brief Intelligence Test (KBIT) (8 control; 8 dyslexic) or the Wechsler Abbreviated Scale of Intelligence (WASI) (5 control; 3 dyslexic) for Performance IQ; and either the CTOPP Rapid Letter Naming (10 control; 7 dyslexic) or the RAN/RAS Letter subtest (3 control; 4 dyslexic)* [*108*] *. Rapid Letter Naming scores are based on z scores to allow for comparison between different subtests used.*

### Visual Stimulus and Shape Extraction Task

**Figure 1:**
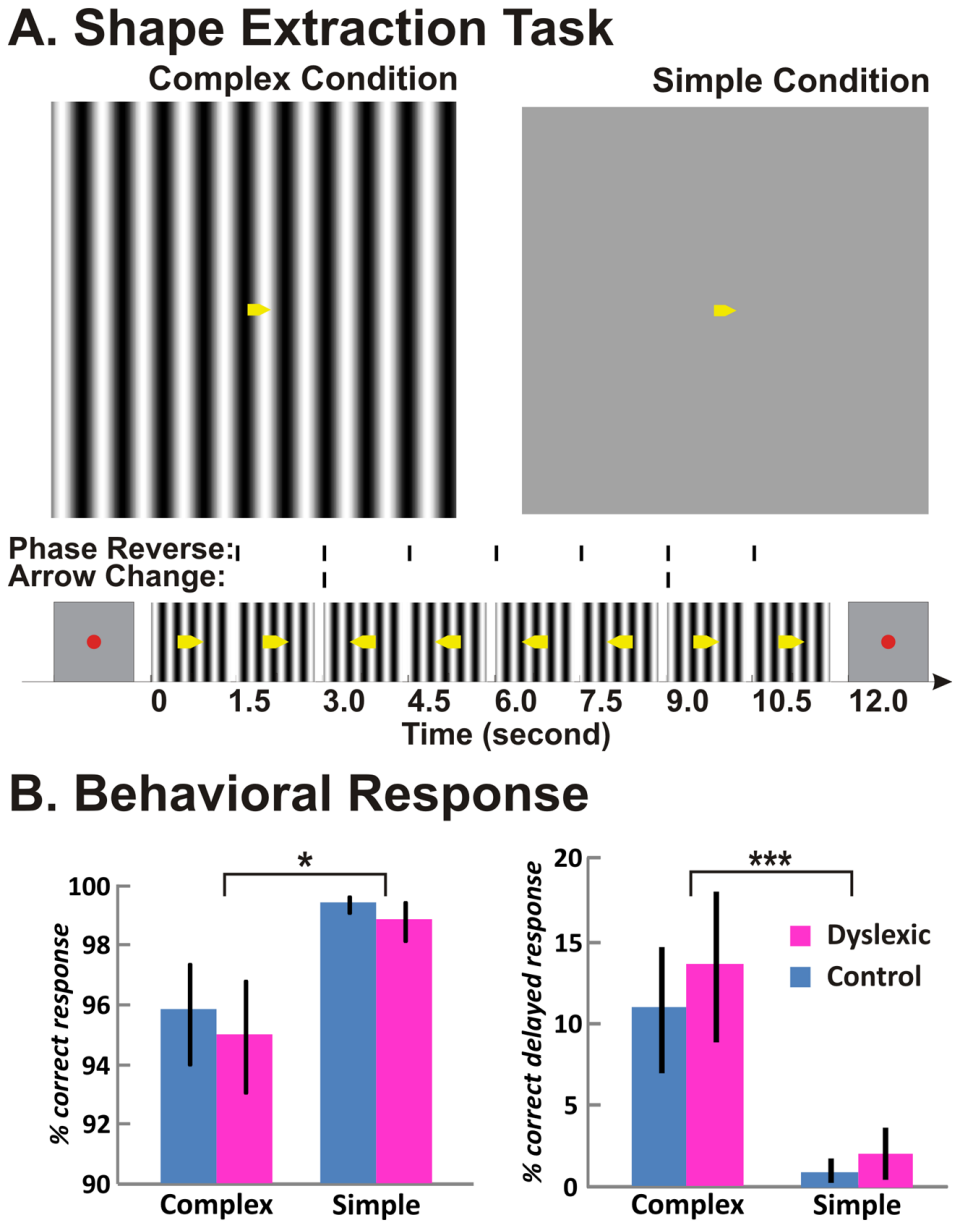
Task and performance. (***A***) The shape extraction task was performed under the complex condition with a large grating background (left panel) and the simple condition with a uniform gray background (right panel). The task had a 3-second fixation period and a 12-second block of stimulus period. During the stimulus period, participants were instructed to push the response button as soon as they saw a change in arrow direction. (***B***) The control and dyslexic groups performed similarly on the shape extraction task. The left panel shows that, for both groups, response accuracy was significantly lower for the complex condition than for the simple condition. The right panel shows that the percent of delayed, but correct, responses was significantly greater for the complex condition than for the simple condition. * *p*<0.01, ** *p*<0.005. Error bars indicate SEM within group.

Visual stimuli consisted of a small arrow displayed at the center with either a background of a uniform gray display (simple condition) or a background of vertical gratings (complex condition) (Fig. 1A). The size of the center arrow was 0.47×0.95°; it was bright yellow with the red and the green luminance values at their maximal luminance. The background during the complex condition was a 0.47 cycle/degree, 100% contrast sinusoidal grating; it was 180° phase reversed every 1.5 seconds (0.33 Hz). The mean luminance of the gratings matched the luminance value of the gray background; the mean luminance values of the gray background and the mean luminance value of the gratings remained constant throughout the experiment. For gratings, the mean luminance value was 3.3 cd/m^2^ with maximum and minimum luminance of 4.2 and 2.4/m^2^, respectively. The low luminance level was used so as to be comparable to previous studies of basic visual perception in dyslexia [18], [35]. Visual stimuli were programmed in Matlab using the Psychophysics Toolbox [36], [37]. Visual stimuli were back-projected from an LCD projector on to a screen, and viewed through a prism mirror as participants were lying on their backs in the scanner. The screen was placed 1.2 meters away from the participants’ eyes; the display subtended a visual angle of 17.8×17.8° and fully covered the opening of the scanner with some corners being concealed. The visual stimuli were simultaneously displayed on a PC monitor in the MRI control room so that they could be monitored continuously by the experimenter.

The shape extraction task required participants to detect the directional change of the arrow. Under both simple and complex conditions, participants were instructed to press a button as soon as they perceived the arrow changing direction, either from left to right or from right to left. Both complex and simple conditions were conducted in blocks of 12 seconds, with a 3-second fixation period between blocks during which participants were asked to look at a small red fixation dot displayed at the center of the screen. There were sixteen blocks per condition. The simple and complex conditions were pseudorandomized and counter-balanced. While there were four additional conditions not reported here, the sequences of the experimental conditions were the same for all participants. Arrow changes occurred randomly for durations in units of three seconds and there were three possible changes within each block of twelve seconds. The timing of the arrow change was synchronized with the phase reversal of the background grating as well as the onset of the time of repetition (TR time) in fMRI scanning. For the simple condition, there were 20 changes of arrow direction for the entire experiment duration: two blocks with no arrow change, ten with one arrow change, two with two changes and two with three changes. For complex condition, there were 28 changes of arrow direction: two blocks with no arrow change, four with one arrow change, six with two changes and four with three changes. During the complex condition, there were 112 phase reversals occurring periodically once every 1.5 seconds so that 25% of the phase reversals coincided with a change in arrow direction. Behavioral responses were recorded on the same laptop computer that was used for the stimulus presentation. The temporal resolution for behavioral responses was 1.5 seconds, the same as the TR. A response was considered correct if the button was pressed within a 3-second window after the arrow changed direction; a response was considered delayed but correct if the button was pressed between 1.5 and 3 seconds after the arrow changed direction.

### Image Acquisition

MRI scans were performed on a 3T Siemens Magnetom Trio Tim system located at the Martinos Imaging Center at MIT. A T2*-weighted gradient-echo echo planar imaging (EPI) sequence was used for fMRI scans; slice thickness = 4 mm, in-plane resolution = 3.1×3.1 mm, repetition time/echo time/flip angle = 1,500 ms/30 ms/90° and the field of view of 200×200 mm^2^. For all participants, a total of 128 images were collected for each experimental condition. At the beginning of each MRI experiment, high resolution (1.3×1.0×1.3 mm) anatomical images were also collected using a T1-weighted, three-dimensional gradient-echo sequence.

### fMRI Data Analysis

Statistical parametric mapping software (SPM5) was used for image pre-processing and statistical analyses (Wellcome Department of Cognitive Neurology, London, http://www.fil.ion.ucl.ac.uk/spm). Functional images were realigned and normalized to an EPI template based on the ICBM152 stereotactic space (an approximation of canonical space). They were re-sampled into 2×2×2-mm cubic voxels, and spatially smoothed by an isotropic Gaussian kernel (4 mm full width at half-maximum). The SPM5 canonical Hemodynamic Response Function (HRF), composed of two gamma functions, was used to predict responses. The predictor was obtained by convolving a time series of step function (box car) with this canonical HRF.

For second level within-group analysis, one sample t-test on contrast images (complex>simple) from first level images were computed separately for dyslexic and control groups. The voxelwise threshold was first set at *p*<0.01 uncorrected, and small volume corrected for multiple comparisons at level of *p*<0.05 for clusterwise correction for occipital, frontal and parietal lobes, respectively. For second-level between-group analyses, two sample t-tests on the contrast images (complex>simple) from first level images were computed. The statistical threshold for between-group contrasts was the same as the single group analysis (*p*<0.01, and *p*<0.05, small volume cluster corrected for occipital, frontal and parietal lobes). The masks used for occipital, frontal and parietal lobes were obtained from Talairach Daemon database atlases [38] in WFU PickAtlas [39].

**Figure 2:**
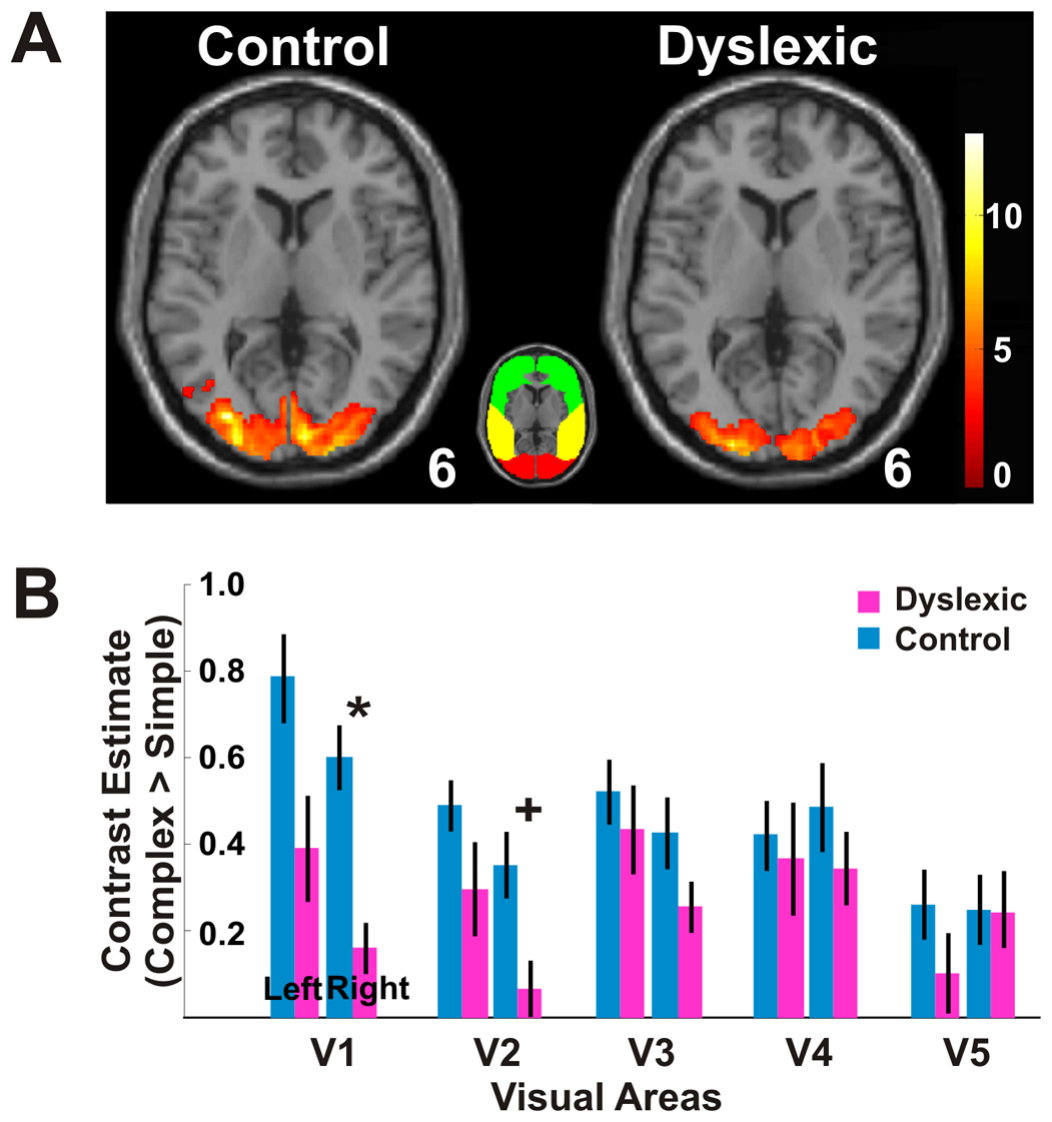
Activations in sensory regions. (***A***) The brain images illustrate occipital activation for control and dyslexic groups. For each image, the transverse slice shows voxels that exhibited a significantly greater activation for the complex condition than for the simple condition (Complex>Simple, *p*<0.05, cluster corrected for occipital lobe). The color bar depicts *t* values, and the color inset illustrates the subdivisions of occipital (red), temporal (yellow), and frontal (green) lobes. The number at the lower right corner of the transverse sections indicates that the images are taken at 6 mm above the AC–PC level. (***B***) Anatomically determined ROI analysis shows greater activation in visual areas V1, V2, V3, V4 and V5 for the control group (bars in blue) than for the dyslexic group (bars in red). ^+^ *p*<0.10, *p<0.05, Bonferroni corrected t-test between groups. Error bars represent SEM within group.

**Figure 3:**
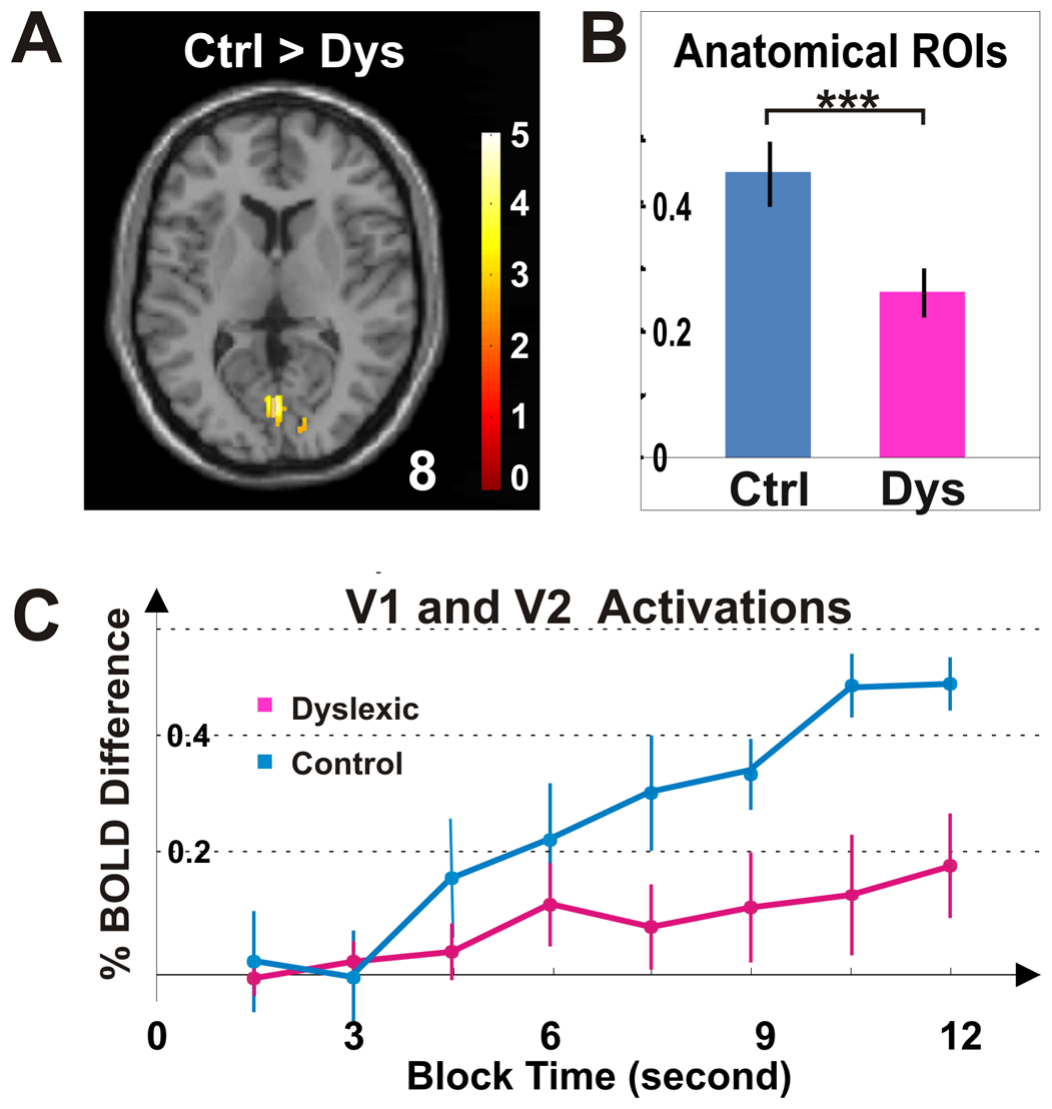
Hypoactivation for dyslexia in sensory regions. (***A***) The brain image illustrates occipital voxels that exhibited significantly greater activation for the control group than for the dyslexic group (Control>Dyslexic, *p*<0.05, cluster corrected for occipital lobe, shown at 8 mm above the AC-PC level). The difference in group activations was most evident at the posterior occipital lobe, around visual areas V1 and V2. (***B***) Occipital activation for the control group was significantly greater than for the dyslexic group. (***C***) The activation time course for anatomically determined visual areas in V1 and V2 shows greater occipital activation for the control group than for the dyslexic group. *** *p*<10^−6^; main effect of group in two-way ANOVA for participant group and visual areas. Error bars indicate SEM within group.

**Figure 4:**
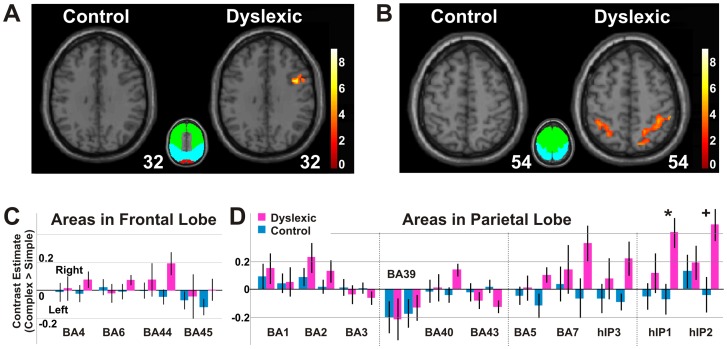
Activations in fronto-parietal regions. (***A***) Brain images illustrate frontal activations for control and dyslexic groups (Complex>Simple, *p*<0.05, cluster corrected for frontal lobe). There is one activation cluster for the dyslexic group, and none for the control group. (***B***) Brain images for parietal activations (Complex>Simple, *p*<0.05, cluster corrected for parietal lobe) indicates that in contrast to bilateral activation for the dyslexic group, there is none for the control group. In both **A** and **B**, the color bar depicts *t* values, and the color inset illustrates the subdivisions of occipital (red), frontal (green) and parietal (cyan) lobes. The transverse images are shown at 32 and 54 mm above the AC-PC level, respectively. (***C***) Anatomically determined ROI analysis shows activations for control and dyslexic groups. (***D***) Anatomically determined ROI analysis shows that the activation of area hIP1 in the dyslexic group is significantly greater than in the control group. From left to right, the parietal areas are shown in four groups: precentral gyrus (BA1, BA2, and BA3), inferior parietal lobule (BA39, BA40, and BA43), superior parietal lobule (BA5, BA7 and hIP3), and intraparietal sucus (hIP1 and hIP2). ^+^ *p*<0.10; * *p*<0.05; Bonferroni corrected t-test. Error bars represent SEM within group.

**Figure 5:**
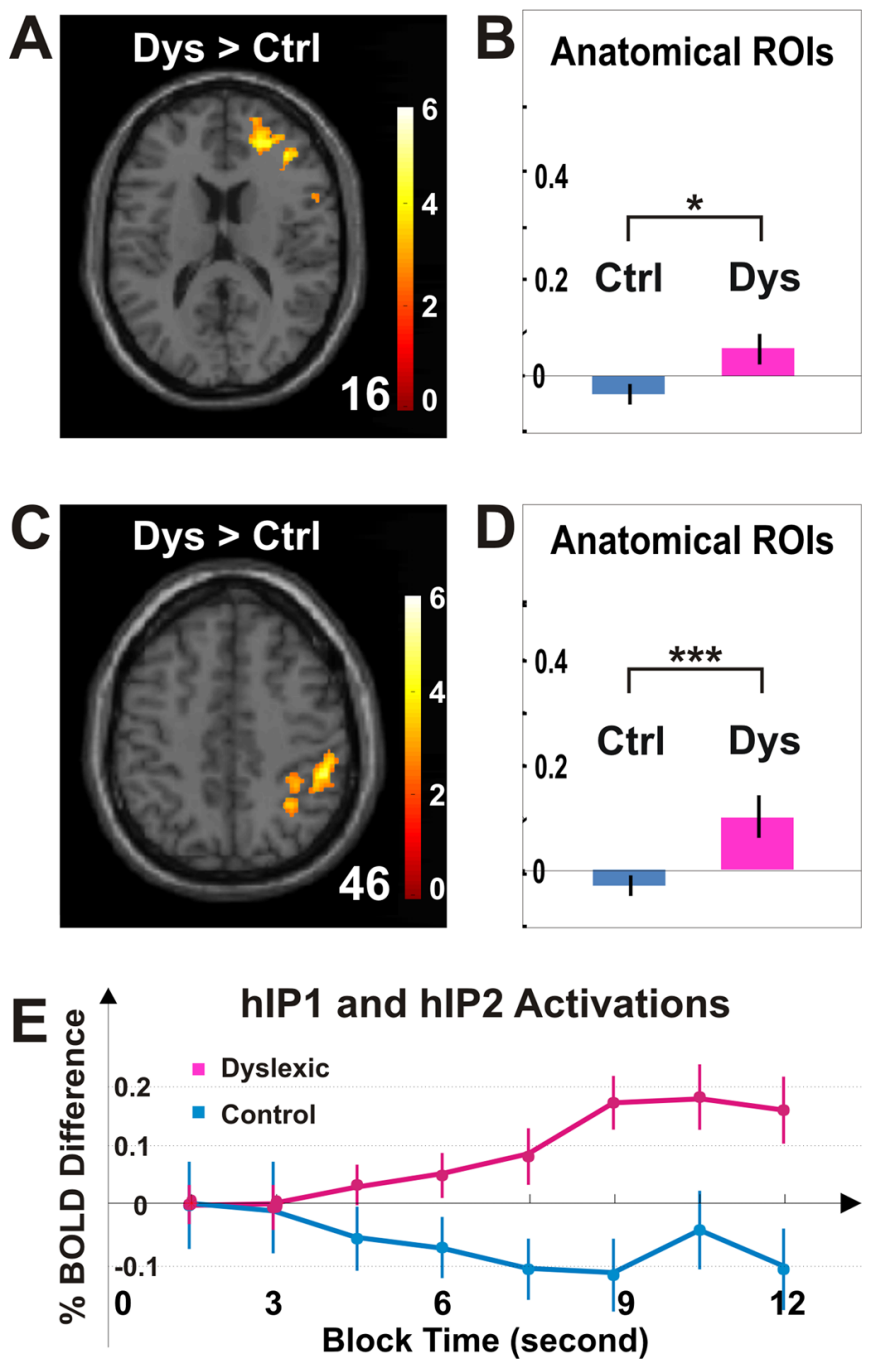
Hyperactivation for dyslexia in fronto-parietal regions. (***A***) The brain image illustrates frontal voxels that exhibited significantly greater activation for the dyslexic group than for the control group (Dyslexic>Control, *p*<0.05, cluster corrected for frontal lobe, shown at 16 mm above the AC-PC level). (***B***) Anatomically determined ROI analysis shows that frontal activation for the dyslexic group was significantly greater than for the control group. (***C***) The brain image illustrates parietal voxels that exhibited significantly greater activation for the dyslexic group than for the control group (Dyslexic>Control, *p*<0.05, cluster corrected for parietal lobe). (***D***) Parietal activation for dyslexic group was significantly greater than the control group. (***E***) The activation time course for anatomically determined areas hIP1 and hIP2 showed greater parietal activation for the dyslexic group than for the control group. * *p*<0.05; *** *p*<10^−6^; main effect of group in two-way ANOVA for group and area. Error bars indicate SEM within group.

All regions of interests (ROIs) used in the anatomical ROI analysis (Fig. 2B, 3B–C, 4C–D, 5B and 5D–E) were determined from cytoarchitectonically identified regions defined from the analysis of human post-mortem brains [38], [39], [40]. Specifically, the different cortical areas such as V1 and V2 were created from the anatomically normalized probabilistic cytoarchitectonic maps, a three-dimensional map in stereotaxic coordinate (the SPM Anatomy Toolbox, http://www.fz-juelich.de/inm/inm-1/spm_anatomy_toolbox, database: AllAreas_v16_MPM.mat). For every ROI, a binary mask was first made through the function of Create-Anatomical-ROIs in the SPM Anatomy Toolbox and all ROI analyses were then performed within this mask (http://www.fz-juelich.de/inm/inm-1/spm_anatomy_toolbox, database: AllAreas_v16_MPM.mat). In this study, the terms of visual areas V1 and V2 were interchangeably used with BA17 and BA18 [41]. V3, V4, and V5 [42], [43], [44], [45] were used with the same naming system as shown in the toolbox. For regions in inferior parietal lobule, BA39 consisted of PGa and PGp; BA40 consisted of PFt, PF, PFm, Pfop, PFcm, OP1 and OP2; and BA43 consisted of OP3 and OP4 [46], [47], [48], [49], [50]. For regions in the superior parietal lobule, BA5 consisted of 5Ci, 5L, 5M, and BA7 consisted of 7A, 7M, 7P, and 7PC and hIP3 [51], [52]. The intraparietal sulcus consisted of hIP1 and hIP2 [51], [52], [53].

**Figure 6:**
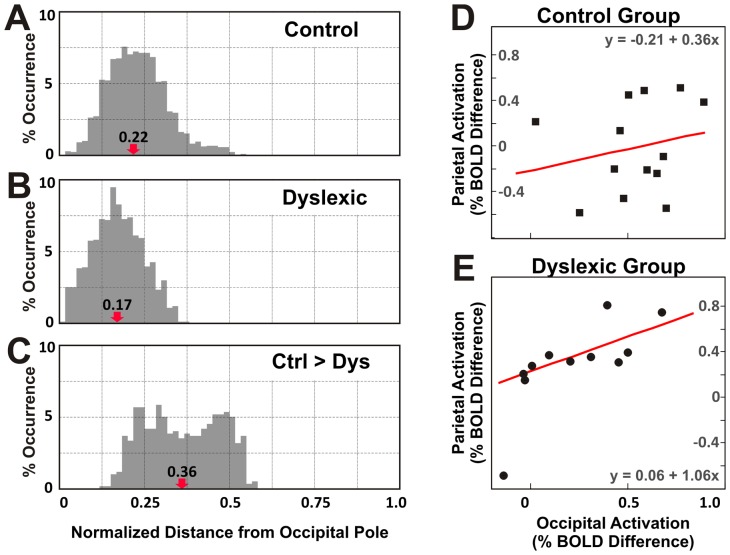
Occipital activation and its relation to parietal activation. (***A***) Frequency histogram shows the distribution of calcarine activation for the control group, along the posterior and anterior direction. The x-axis shows the distance between the intersected coronal section and the occipital pole, normalized to maximal length of calcarine fissure along the posterior and anterior direction. The y-axis shows the percent occurrence of activation within a coronal section over all activation within the calcarine structure. The red arrow indicates the median distance and the number above shows the value of the median distance. (***B***) The distribution of calcarine activation for the dyslexic group is similar to the one for the control group. (***C***) The distribution of dyslexic calcarine hypoactivation differs from A or B: it evades areas around the occipital pole. (***D***) For participants in the control group, parietal activations in areas hIP1 and hIP2 were not not correlated with occipital activations in areas V1 and V2 (N = 13, R^2^ = 0.015, and p>0.05). The line in red shows the linear regression line. (***E***) In contrast, for participants in the dyslexic group, the parietal activations were positively correlated with occipital activations (N = 11, R^2^ = 0.526, and p<0.05).

The ROI images and the patterns of activation were reviewed visually, and presented through xjview8 (viewing program for SPM, Xu Cui, http://www.alivelearn.net/xjview/). For anatomically determined ROI analysis, the contrast estimates and the activation values were extracted with custom-made software rex.m (Susan Whitfield-Gabrieli, http://web.mit.edu/swg/software.htm) (Figs. 3B, 5B and 5D). For statistical analysis, the two-way ANOVAs, the two-tailed *t*-*tests* for groups with unequal variance, and the linear regression (Figs. 6D and 6E) were performed with custom-made Matlab scripts (MathWorks, Natick, MA). Bonferroni corrections were used to address the problems of multiple comparison [54] (Figs. 2B, 4C and 4D). With a threshold of p_threshold_ and given n pairs of tests conducted, we would obtain an adjusted *threshold* of p_adjust_ = p_threshold_/n. Respectively, the significance levels were adjusted to 0.005 for occipital (0.05/10, n = 10), 0.00625 for frontal (0.05/8, n = 8) and 0.00227 for parietal (0.05/22, n = 22) lobes.

For time course extractions, the mean BOLD signal in each ROI from the functional image time series was extracted with custom-made software rex.m; the difference in BOLD signal between simple and complex conditions was then used to evaluate the responses for shape extraction within the block time with values at each TR. To further normalize the results for all participants, a grand average value of Resp_avg_all_ was calculated by averaging the responses at the two fixation points between different experimental conditions for all participants included in this study. Respectively for dyslexic and control groups, the percentage BOLD difference shown in Figs. 3C and 5E was then calculated as BOLD_difference_group_(t) = [Resp_group_(t)-Resp_avg_all_]/Resp_avg_all_*100%.

To examine the distribution of V1 activation along cortical representation of the visual field, we assumed that the retinotopic map of V1 was laid out across the folded cortical surface in the gray matter of the calcarine fissure. The central (foveal) part of the visual field is represented most posteriorly at the occipital pole, whereas the most peripheral regions of the visual field are located forward at the most anterior part of the calcarine fissure. To calculate the location of occipital activation along anterior-posterior direction of the calcarine fissure, we first obtained a mask of calcarine fissure from AAL atlases [55] in WFU PickAtlas [38] and found its most anterior and posterior locations. The most anterior point of calcarine structure was defined as the point with the largest y-value, and the most posterior point was the one with the smallest y value. We then divided the 3D structure of calcarine fissure into evenly distributed 1 mm thick coronal slices and calculated the number of activated voxels within the calcarine fissure mask.

## Results

### Behavioral Profile of Participants

The control and dyslexic groups performed similarly on measures of non-verbal cognitive ability (Table 1). The dyslexic group performed significantly worse than the control group for all reading measures for words and pseudowords under both untimed and timed conditions (i.e., for both accuracy and speed). The dyslexic group also performed significantly slower on the rapid letter naming task.

### Shape Extraction Task and In-Scanner Performance

For response accuracy (% correct), there were reliable differences between conditions, but not for groups or for any interaction (Fig. 1B, left panel; two-way ANOVA for condition, group and interaction between condition and group, F_condition_(1,42) = 7.68, *p*<0.01; F_group, interaction_(1,42) = 0.22, 0.01; *p* values >0.05). For response latency, we examined the rate of delayed but correct reaction times when the correct responses occurred not within but after 1.5s, and before the end of subsequent TR time. Similar to the response accuracy, response latencies were significantly longer for the complex than for the simple condition (Fig. 1B, right panel; main effect of condition, F(1,42) = 11.79, *p*<0.005). There was neither a main effect of group nor an interaction (F(1,42) = 0.36, 0.06; *p* values >0.05). These results indicated that, for both dyslexic and control groups, identifying the arrow change from the grating background at a low luminance were more difficult than from the uniform background at the same luminance level, but there was no difference behaviorally between the two groups on this simple perceptual task (Fig. 1B).

### Hypoactivation in Sensory Regions in Dyslexia

For both groups, there was greater bilateral activation for the complex than for the simple condition in the occipital lobe (Fig. 2A and Table 2; Complex>Simple, *p*<0.05, cluster corrected for occipital lobe). For both groups, occipital activations included visual areas Brodmann area 17 (BA17), BA18, BA19, and cuneus (Table 2). The extent of occipital activation in the dyslexic group was 40.0 cm^3^, somewhat smaller than the control group of 47.1 cm^3^ (Table 2). Direct comparisons between the groups revealed significantly greater activation for the control than for the dyslexic group in occipital regions (Fig. 3A and Table 3; Control>Dyslexia, *p*<0.05, cluster corrected for occipital lobe). In contrast, no occipital region showed greater activation for the dyslexic than for the control group (Table 3; Dyslexia>Control, *p*<0.05, cluster corrected for occipital lobe).

**Table 2:** Activation for Dyslexic and Control Groups.

		Control Group (Complex>Simple)							Dyslexic Group (Complex>Simple)					
	Cluster	Voxel					Cluster		Voxel					
	p_cor	*k*	*(x y z ) Z Score* Brain Region				p_cor		*k*	*(x y z ) Z Score* Brain Region				
**Occipital**	0	5883	−30 −84 8	*5.73*	MOG	BA18		0	4995	−20 −98 6	*5.28*	MOG	BA18,	
			8 −94 −6	*5.56*	Cuneus	BA19				−24 −98 −6	*5.08*	Cuneus	BA19	
			2 −80 −2	*5.45*	LinG	BA17				−26 −94 0	*5.01*	LinG	BA17	
**Frontal**								0	396	46 6 32	*4.66*	PrecentralG	BA6	
										52 4 34	*4.15*	IFG	BA9	
										50 28 18	*3.47*	MFG		
**Parietal**								0	1096	58 −22 32	*4.33*	IPL	BA7	
										50 −42 50	*3.96*	SupMG	BA40	
										30 −56 62	*3.77*	PostcentralG	BA2	
								0	298	−26 −54 58	*3.77*	IPL	BA40	
										−36 −44 46	*3.18*	PostcentralG	BA7	
										−40 −44 56	*3.1*	SPL		

**Table 3:** Differential Activation between Groups.

		Hypoactivation (Control>Dyslexic)						Hyperactivation (Dyslexic>Control)				
	Cluster	Voxel					Cluster	Voxel				
	p_cor	*k*	*(x y z ) Z Score* Brain Region				p_cor	*k*	*(x y z ) Z Score* Brain Region			
**Occipital**	0	494	2 −78 6	*3.72*	Cuneus	BA18						
			2 −80 −2	*3.71*	Calcarine	BA17						
			10 −86 −2	*3.18*								
**Frontal**							0	404	40 6 32	*4.22*	IFG	BA9
									46 4 32	*4.12*	PreCentralG	BA44
									52 4 36	*3.42*	MFG	
							0	418	24 48 20	*3.99*	SFG	BA10
									38 30 22	*3.98*	MFG	BA46
									38 36 18	*3.77*	IFG	
**Parietal**							0	783	60 −22 30	*4.57*	IPL	BA40
									54 −24 32	*4.46*	SupMG	BA2
									50 −38 44	*3.94*	PostcentralG	

p-cor values are corrected for multiple comparisons at the level of occipital, frontal and parietal lobes (except for Z Scores reported in italics). *k* is the number of voxels in each cluster. (x y z) represents the location of maximal response in MNI coordinates. The brain regions listed are obtained from the Talairach Daemon database, and they are the first three largest brain areas that are at or above 10% of the total area. F = Frontal; G = Gyrus; I = Inferior; L = Lobule; Lin = Lingual; M = Middle; O = Occipital; P = Parietal; S = Superior; and SupM = SupraMarginal.

For the dyslexic group, anatomically determined ROI analysis showed all activation values examined in the left and right visual areas V1, V2, V3, V4 and V5 were below the values in the control group (Fig. 2B). The average value of activation for the dyslexic group was significantly lower than the control group (Fig. 3B; two-way ANOVA of group and area, F_group_(1,220) = 23.89, *p*<10^−6^, F_area_(9,220) = 3.99; *p<*0.0005 and F_interaction_(9,220) = 1.38, *p*>0.05). The activation time course extracted from anatomical ROIs in occipital V1/V2 further verified the effect of dyslexic hypoactivation in visual sensory areas as indicated by mean differences in activation between conditions by TR (Fig. 3C).

### Hyperactivation in Frontal and Parietal Lobes in Dyslexia

In contrast to occipital hypoactivation for the dyslexic group, we also found hyperactivation in both frontal and parietal lobes (Figs. 4, 5 and Table 2). In the frontal lobe, there was greater and more widespread activation in the dyslexic group than in the control group (Fig. 4A and Table 2; 3.2/0.0 cm^3^; Complex>Simple, *p*<0.05, cluster corrected for frontal lobe). For the control group, there was no activation found in the frontal lobes (Fig. 4A, left panel and Table 2). For the dyslexic group, activation in precentral, middle frontal, and inferior frontal gyri was found in the right hemisphere (Fig. 4A, right panel and Table 2). Direct comparisons between the groups revealed significantly greater activation for the dyslexic than the control group (Fig. 5A and Table 3; Dyslexia>Control, *p*<0.05, cluster corrected for frontal lobe). In comparison to frontal activations for dyslexic group alone (Fig. 4A), the hyperactivation obtained from the group contrast between the dyslexic and control participants included an additional cluster located more towards the anterior and ventral portion of the frontal lobe (Fig. 5A).

In the parietal lobe, there was bilateral activation in the dyslexic group, but no above-threshold activation in the control group (Fig. 4B and Table 2; 11.2/0.0 cm^3^; Complex>Simple, *p*<0.05, cluster corrected for parietal lobe). The activation for the dyslexic group was bilateral, but substantially greater in the right than in the left hemisphere (Table 2; 8.8/2.4 cm^3^). Greater activation in the dyslexic group than in the control group occurred in the right hemisphere; primarily in the inferior parietal lobule (Fig. 5C and Table 3; Dyslexia>Control, *p*<0.05, cluster corrected for parietal lobe). In contrast, no frontal or parietal region showed greater activation for the control than for the dyslexic group (Table 3; Control>Dyslexia, *p*<0.05, cluster corrected for frontal or parietal lobe).

Anatomically defined regions of interest (ROIs) analysis were used to further quantify the findings of increased frontal and parietal activation in dyslexia. The dyslexic group showed significantly greater activation than the control group in frontal regions (Figs. 4C and 5B; BA4, BA6, BA44, and BA45; two-way ANOVA, F_group_(1,176) = 4.46, *p*<0.05; F_area_(7,176) = 0.74, *p*>0.05; and F_interaction_(7,176) = 0.61, *p*>0.05) and parietal regions (Figs. 4D and 5D; BA1, BA2, BA3, BA5, BA7, BA39, BA40, BA43, and hIP1, hIP2, hIP3; two-way ANOVA, F_group_(1,484) = 16.82, *p*<10^−6^; F_area_(21,484) = 2.21, *p*<0.005; and F_interaction_(21,484) = 1.55, p>0.05). The parietal hyperactivation for the dyslexic group was further confirmed by the activation time course extracted from parietal hIP1/hIP2 (Fig. 5E).

### Pattern of Occipital Hypoactivation and Relation between Occipital and Parietal Activations in Dyslexia

The V1 activations distributed along the cortical representation of the visual field were measured by dividing the calcarine fissure into evenly distributed fine coronal slices (subdivisions along anterior/posterior dimension in the 3D volume) and counting the number of above-threshold voxels of activation. In unit of the maximal length of calcarine fissure along the anterior/posterior direction, activation for the control group extended from 0.03 to 0.55, with a median distance of 0.22 from the most posterior end of the cortex, the occipital pole (Fig. 6A). The distribution for the dyslexic group extended from 0.02 to 0.38, with a median distance of 0.17 (Fig. 6B). Unlike the activation distribution for the control or dyslexic groups, the occipital hypoactivation extended more anteriorly from 0.13 to 0.58, with a median distance of 0.36 (Fig. 6C). The dyslexic hypoactivation occurring in visual cortex appeared to occur in more anterior calcarine cortex, likely representing peripheral visual areas, and to reflect a deactivation (more activation for the simple than the complex conditions) in the dyslexic group (Fig. 6A–C).

To understand the relation between occipital hypoactivation and parietal hyperactivations in the dyslexic group, linear regression analysis between occipital and parietal activations were performed for the dyslexic and the control groups respectively. There was no significant correlation between occipital V1/V2 activation (values for individual participants in Fig. 3B) and parietal hIP1/hIP2 activation (values for individual participants in Fig. 5D) in the control group (Fig. 6D, N = 13, R^2^ = 0.015, and *p*>0.05). In contrast, occipital activations were significantly correlated with the parietal activations in the dyslexic group (Fig. 6E, N = 11, R^2^ = 0.526, and *p*<0.05).

## Discussion

We used fMRI to examine the underlying neural mechanisms for visual shape extraction from a complex relative to a simple visual background, and found occipital hypoactivation accompanied with fronto-parietal hyperactivation in adults with persistent dyslexia. The brain organization for basic visual shape extraction from a complex background appears to be fundamentally different in people with or without dyslexia. Because this atypical balance of activation occurred in a simple visual task without the involvement of reading or language, these findings support the view that visual differences, in addition to well-documented phonological differences, contribute to reading difficulty, on average, in dyslexia. Prior neuroimaging studies of basic visual processing had examined motion and only reported decreased posterior activations in dyslexia (18, 19), but here we found differences related to shape extraction. These differences involved opposite patterns of atypical activation in dyslexia with decreased activation in posterior visual areas associated with basic visual processing and increased activation in fronto-parietal areas associated with attentional and cognitive processes. The regions of increased activation suggest a mechanism by which weaknesses in lower-order perceptual cortices may interfere with reading-relevant processes in higher-order cortices.

### Shape Extraction Task and Behavioral Performance in Dyslexia

In this experiment, we chose the term “shape extraction” to contrast with prior imaging studies of basic visual processing in dyslexia that focused on motion, and to offer a relatively theoretically neutral term describing the task demand. Following the notion that “the human visual system efficiently extracts and identifies shapes and forms, including letters, digits, signs, and line drawings” [56], we have conceptualized the primary task contrast as one of shape extraction rather than shape identification. Participants had to separate the shape of the arrow from the background grating in one condition but not the other, but identifying the altered shape of the simple arrow was constant across conditions.

The task of shape extraction involves both foveal processes related to analysis of the arrow and peripheral processes related to the full screen background. The finding that group differences were greatest in calcarine cortex associated with peripheral rather than foveal visual processing suggests that the hypocativation in dyslexia was not simply stimulus-driven by the arrow. Instead, the hypoactivation may reflect processes that integrated the effect of the task, the full screen background, and the processing of the target arrow.

The brain differences for individuals with dyslexia occurred despite no group differences in task performance. On the one hand, this means that the brain differences were not a consequence of or secondary to behavioral difficulties; on the other hand, functional brain differences are of interest primarily to the extent that they are relevant to behaviors, such as reading. One possible interpretation relates to the ease of the task on which most participants performed nearly perfectly. On such an easy task, individuals with dyslexia may have been able to fully compensate behaviorally, and the frontoparietal hyperactivation for dyslexia may reflect the recruitment of additional neural resources that supported such compensation. It is also possible that the limited temporal resolution of our behavioral measure obscured subtle group differences in latency. In either case, there is evidence from other studies with non-verbal tasks that individuals with dyslexia tend to exhibit behavioral deficits as a function of task difficulty for both verbal [57], [58] and non-verbal stimuli [21], [59], [60], [61], [62], [63]. Therefore, it may be that the minimal challenge of the task in the present study revealed functional brain differences that could lead to impaired behavioral performance in more demanding experimental conditions or during the reading of letters in the context of other letters or words. Future studies can examine conditions under which individuals with dyslexia perform both similarly and also less well than typical readers to further relate the patterns of brain activation to intact and impaired visual performance in dyslexia.

### Interpretations for Occipital Hypoactivation and Fronto-Parietal Hyperactivation

Occipital hypoactivation accompanied with fronto-parietal hyperactivation in dyslexia demonstrates that both sensory and attentional visual processes may be altered in dyslexia. These findings may be interpreted in several ways because sensory and attentional processes interact in many ways [64], [65], [66], [67], [68], [69], [70]. Occipital hypoactivation and fronto-parietal hyperactivation could reflect separable visual sensory and attentional deficits in dyslexia. Alternatively, occipital hypoactivation may cause fronto-parietal hyperactivation in dyslexia. Typical readers may be able to extract visual shape information based largely on early subcortical and cortical processes. Greater activation of early visual areas in typical readers may reflect this active visual shape extraction processing that alleviates the need for higher-order cortices to support focused attention on this relatively simple task. In contrast, reduced activation of early visual areas in readers with dyslexia may signal impaired visual perceptual process that causes the recruitment of compensatory attentional resources supported by fronto-parietal regions. The idea that higher-order brain regions are recruited when lower-order regions cannot support perception of stimuli is supported by studies reporting greater activation in fronto-parietal regions when sensory information is degraded either by presenting degraded words to typical readers or the blurring of vision in patients with macular degeneration [71], [72].

The finding that occipital activation was significantly correlated with parietal activation in the dyslexic group, but not in the typically reading group, suggests that the atypical occipital and parietal activations were associated rather than dissociated in dyslexia. Perhaps greater top-down attentional processes supported by parietal cortex enhanced, or attempted to enhance, bottom-up visual processes supported by occipital cortex.

### Magnocellular Pathway Hypothesis of Dyslexia

Visual deficit hypotheses for dyslexia can be divided broadly into two categories that focus on either bottom-up sensory deficits or top-down attentional problems. Among the sensory hypotheses, the most studied is the magnocellular visual pathway hypothesis positing that dyslexia is partially due to a disorder of the fast processing magnocellular (M) sub-system, a visual pathway extending from the retina to the occipital and parietal areas of the brain. The magnocellular hypothesis of dyslexia has also been extended to proposals of weaknesses in functionally analogous auditory and tactile modalities [73], [74], [75], [76], [77]. Because the visual stimuli in this study were not designed to selectively involve the magnocellular system, our findings cannot directly support or challenge the magnocellular hypothesis of dyslexia. Our finding of hypoactivation for the dyslexic group in sensory regions is consistent with previous neuroimaging studies reporting reduced activations to moving stimuli in early visual areas [18], [19], [78]. The present study extends those prior findings in two ways. First, those studies examined brain responses to visual motion, whereas we examined brain responses to stationary visual shape extraction. Second, prior studies focused on verifying the magnocellular hypothesis in early visual areas, and did not examine the frontal and parietal regions associated with visual attention. The present study, therefore, demonstrates that altered brain activations in dyslexia are not limited to early visual areas involved in visual motion, but rather are accompanied by altered responses in higher-order cortical regions as well.

### Noise-exclusion Hypothesis and Visuo-spatial Attention Hypothesis in Dyslexia

Instead of emphasizing bottom-up sensory deficits, the noise-exclusion and the visuo-spatial attention hypotheses of dyslexia focus on deficits in top-down cognitive processes. Psychophysical studies have reported that individuals with dyslexia have a deficit for perceiving simple non-verbal stimuli presented in white noise, in both vision and audition [16], [26], [27], [28], [29], [79], [80], [81]. There has not been prior evidence about a potential brain basis for the noise exclusion deficit in dyslexia. Because the complex condition in the current study can be considered as the extraction of arrow shape information from the background noise of a grating, our results suggest that the underlying neural mechanism for noise exclusion in dyslexia could be that weakened early visual processes fail to discriminate useful signals from background noise, and such weakened sensory processes are compensated for by the recruitment of higher-order attentional processes.

We found parietal hyperactivation that may be associated with the visuo-spatial attention hypothesis of dyslexia. The visuo-spatial attention hypothesis of dyslexia has been motivated by behavioral studies reporting impairments on visual attentional tasks, such as identifying a target that was spatially or temporally distinguished from distractors in the background or masks [21], [22], [23], [24], [82], [83], [84]. In the brain, the effect of visual attention is often associated with activations in fronto-parietal regions [85], [86], [87], [88], [89], [90], [91], [92], [93], [94], [95]. The attentional hypothesis of dyslexia is supported by the present finding of hyperactivation in frontal-parietal regions and a prior finding of parietal hypoactivation [25]. The difference in hyper- vs. hypoactivation in the parietal lobe may be due to differences in participant selection, visual stimuli involved in tasks, or both. The present study recruited people with poor reading only whereas the prior study recruited people with both reading and visual attention span deficits; thus, the discrepancy in parietal activation might be due to the fact that people with and without attention deficits have different neural mechanisms for attentional modulation. In addition, whereas the present study used simple nonverbal visual stimuli without any involvement of language or reading, the visual stimuli in the prior study consisted of a mixture of geometric shape and letters. Prior studies employing reading-related tasks have shown reduced activation in parietal regions of people with dyslexia [96], [97], [98], so it may be that individuals with dyslexia can compensate via parietal hyperactivation for simple tasks (as in the present study), but not for more demanding reading tasks. Given the evidence of right-hemisphere specialization for visuo-spatial functions from both lesion studies [99] and imaging studies (e.g., [100]), the visuo-spatial nature of the present study may also explain why activation differences between groups were predominantly right-lateralized.

### Limitations of Study

Two limitations of the present study may be addressed in future studies. First, the present study lacked a resting baseline, precluding separate analyses of the complex and simple conditions relative to baseline of fixation. Therefore, we cannot determine if the group differences arose from only the complex or simple conditions or both conditions. A previous study reported no activation difference in inferior occipital-temporal cortex between dyslexic and normal children in a contrast between identifying a shape in isolation versus looking at a blank screen with only a fixation point [25]. This supports the idea that the present difference may arise from the complex condition. A resting or fixation baseline condition, however, may not be definitive, because resting-state functional connectivity appears to differ in typical and dyslexic readers [101], [102]). Second, this study was performed with adults who had a childhood diagnosis of dyslexia and continued to demonstrate behavioural manifestations of dyslexia in adulthood. It is unknown whether this atypical pattern of brain activation for visual shape extraction is present in children with dyslexia, and therefore a potential cause for dyslexia, or whether it emerges through development as a type of adaptation, and therefore is a consequence of dyslexia (e.g., the consequence of far less reading practice). In prior studies, core brain differences observed in adults with dyslexia have resembled brain differences observed in children with dyslexia, such as temporo-parietal hypoactivation for phonological analysis in adults [103], [104] and in children [4], [98], and reduced frontal activation for rapid auditory non-verbal stimuli in adults [4] and in children [105]. These prior similarities between children and adults suggest that a similar atypical balance may be found in children with dyslexia, but only studies conducted with children can determine whether this atypical balance is present before reading or early in reading in children who progress to dyslexia.

### Relation of Atypical Balance of Activation to Reading and Dyslexia

The present study extends knowledge about the neural correlates of visual processing in adult dyslexia in three fundamental ways. First, prior imaging studies of basic visual processing with non-verbal stimuli in dyslexia focused on *motion* (18, 19, 73), and here we focused on *shape extraction*, a process that is important in reading letters and words embedded in text. Second, in addition to prior imaging findings of reduced activations in occipito-temporal brain areas specialized for vision, we found altered brain responses in higher-order brain regions associated with top-down attentional network. Our finding in the higher-order brain regions is consistent with multiple imaging studies of dyslexia employing reading-related tasks [96], [97], [98]. Third, the concurrent and correlated hypoactivation and hyperactivation in different brain regions in a simple visual task in dyslexia raises a possible direct link between lower-order visual weaknesses and higher-order reading weaknesses.

Reading is understood to be an interaction between perceptual processes involved in the extraction of letter shape in the complex visual environment of the printed page and cognitive processes involved in comprehension, interpretation, and prediction. Previous reading studies have demonstrated that phonological recoding involves central attention processes as shown by the effect of other ongoing mental events that also require central attention [106], [107]. Although future studies will be needed to directly link a basic brain alteration of visual shape extraction to the specific processes used to read print, the finding of an atypical balance between occipital and fronto-parietal functions suggests how a difference in visual shape extraction may impede reading through a shared central attentional mechanism. The underengagement of sensory processes supported by early visual areas may fail to support effective shape extraction during reading, and the consequent overengagement of attentional areas may compete for cognitive resources and thus further undermine fluent reading.
